# Mechanisms and Applications of Neuromodulation Using Surface Acoustic Waves—A Mini-Review

**DOI:** 10.3389/fnins.2021.629056

**Published:** 2021-01-27

**Authors:** Danli Peng, Wei Tong, David J. Collins, Michael R. Ibbotson, Steven Prawer, Melanie Stamp

**Affiliations:** ^1^School of Physics, The University of Melbourne, Melbourne, VIC, Australia; ^2^National Vision Research Institute, Australian College of Optometry, Carlton, VIC, Australia; ^3^Department of Optometry and Vision Sciences, The University of Melbourne, Parkville, VIC, Australia; ^4^Biomedical Engineering Department, The University of Melbourne, Melbourne, VIC, Australia

**Keywords:** surface acoustic wave, neuron, neurostimulation, neuromodulation, ultrasound, mechanisms

## Abstract

The study of neurons is fundamental for basic neuroscience research and treatment of neurological disorders. In recent years ultrasound has been increasingly recognized as a viable method to stimulate neurons. However, traditional ultrasound transducers are limited in the scope of their application by self-heating effects, limited frequency range and cavitation effects during neuromodulation. In contrast, surface acoustic wave (SAW) devices, which are producing wavemodes with increasing application in biomedical devices, generate less self-heating, are smaller and create less cavitation. SAW devices thus have the potential to address some of the drawbacks of traditional ultrasound transducers and could be implemented as miniaturized wearable or implantable devices. In this mini review, we discuss the potential mechanisms of SAW-based neuromodulation, including mechanical displacement, electromagnetic fields, thermal effects, and acoustic streaming. We also review the application of SAW actuation for neuronal stimulation, including growth and neuromodulation. Finally, we propose future directions for SAW-based neuromodulation.

## Introduction

Neurological disorders such as Alzheimer’s and Parkinson’s disease, strokes, multiple sclerosis, epilepsy, migraines, and other brain pathologies are the global leading causes of disability-adjusted life-years (the sum of years of life lost and years lived with disability), causing intense discomfort to patients and considerable social and economic burden (Feigin et al., 2017). Despite intense research to overcome these degenerative and life-threatening neurological conditions, the mechanisms behind these disorders are not fully understood. Moreover, most existing therapies only mask the symptoms to mitigate suffering rather than treating the underlying causes (Feigin et al., 2017). Current therapies to treat nervous system conditions include drugs and direct neuronal stimulation using electrical, optical, magnetic, and ultrasound modalities. Ultrasound excitation in particular presents an effective alternative for treating neurological disorders by either mediating drug delivery or directly stimulating neurons (Leinenga et al., 2016).

Ultrasound-based neuromodulation was first introduced in 1929 (Harvey, 1929) and is commonly delivered via traditional ultrasound transducers comprised of piezoelectric materials, e.g., lead zirconate titanate (PZT) where the application of an electric potential results in a mechanical displacement (Menz et al., 2013; Sassaroli and Vykhodtseva, 2016; Yang et al., 2019). The traditional ultrasound transducer is typically actuated in bulk-wave mode resonance often at frequencies in the range of several kHz to a few MHz, with energy coupled from the transducer into adjoining media. However, this actuation mode has several limitations: (1) Resonating bulk acoustic waves inside the piezoelectric materials dissipates parts of the energy as heat, resulting in self-heating of the ultrasonic element. This heating can irritate and even burn the skin, especially when the transducer is operated at high intensity, and also reduces device efficiency (Duck et al., 1989; Calvert and Duck, 2006; Gulick et al., 2017). (2) The dimensions of traditional ultrasound transducers, along with their significant power requirements, hinder their application as wearable or implantable devices for targeted stimulation (Zhou et al., 2019). Finally, (3) native nano/microbubbles in living tissue oscillate with the application of ultrasound at frequencies in the range of several kHz to a few MHz, and can rapidly collapse and lead to shock waves (i.e., inertial cavitation) (Miller et al., 1996; Miller, 2007; Zhong et al., 2011). These cavitation-induced shock waves are dangerous to the neurons and can lead to cell death. This limits the maximum safe operating power, potentially limiting the ability to produce threshold neuromodulation effects.

More recently a different transducer type that has alternate wavemodes, namely surface acoustic waves (SAWs), has been explored for neuromodulation. SAW devices, as compared to bulk wavemode transducers, can produce far higher frequencies (several MHz to GHz) (Shilton et al., 2014). SAWs are specific modes of acoustic waves, which are composed of both longitudinal and transverse components propagating along the surface of a piezoelectric material with an amplitude that decays exponentially into the substrate bulk. Each volume element at the surface moves elliptically in the plane formed by the surface normal vector and the SAW propagating direction (Barnkob et al., 2018). These waves are most commonly generated via an interdigitated transducer (IDT) on the surface, which consists of a set of comb-like electrodes interleaved with another set of parallel electrodes. To generate a SAW, a sinusoidal electrical signal with a frequency *f* is applied to the IDT. The actuation frequency is determined according to the resonant frequency of the device,

(1) $$f=\frac{v}{\lambda },$$

where λ represents the periodic distance between two fingers of the same electrode of the IDT and *v* is the sound velocity of the piezoelectric substrate. Due to the inverse-piezo effect, the electric signal is translated into a mechanical deformation of the surface resulting in an acoustic wave propagating along the surface. When entering a liquid, part of the SAW refracts into the liquid resulting in acoustic streaming at the Rayleigh angle θ_*R*_,

(2) $$\theta_{R}=\sin^{-1}\frac{v_{l}}{v_{s}},$$

where *v_l* and *v_s* are the sound speeds in the liquid and the substrate’s SAW wave speed, respectively. SAWs can take the form of both traveling SAW (TSAW) and standing SAW (SSAW). Whereas a TSAW is generated by a single IDT, with wavefronts propagating along the surface, a SSAW is produced with the addition of a second opposing IDT. The intersection of the counter-propagating wavefronts arising from these IDTs produces time-averaged nodal/antinodal displacements (Collins et al., 2015).

Compared to bulk wavemode transducers, SAW devices have unique advantages. These include (1) less self-heating, (2) miniaturized dimensions, and (3) low cavitation. (1) The tissue heating comes from both local heat dissipation from the acoustic field and the heat conduction from the transducer itself—self-heating. Although the local heat dissipation cannot be controlled, for a given power, a SAW device can produce less self-heating than bulk wavemode transducers, since the SAW energy is confined within the surface of a material. In this case heat dissipation mainly occurs at one wavelength depth from the surface in contrast to traditional ultrasound transducers, where the heat dissipates throughout the entire bulk (Duck et al., 1989; Killingback et al., 2008). (2) SAW devices are fabricated with piezoelectric materials using standard photolithography processes, resulting in transducer dimensions 10^–2^–10^–4^ m, and focused fields spanning widths as small as tens of microns (Collins et al., 2016). (3) Traditional ultrasound transducers typically operate at lower frequencies than SAW devices. High-frequency ultrasound is less likely to generate cavitation effects in cell membranes (Yang and Jo, 2014; Lin et al., 2018). These cavitation effects have been found to trigger cell death by inducing the opening of pores in the cell membrane (Zhong et al., 2011; Wiklund, 2012).

In this article we will discuss the potential mechanisms behind SAW stimulation of neurons, emphasizing the possible effects on the cell membrane, ion channels and cytoplasm, and review previous studies on SAW-based neuromodulation (see Table 1). Finally, we will propose future directions of SAW/neuron interaction studies and applications.

**TABLE 1:** Summary of the acoustic parameters used in stimulating neurons via SAW.

**Study**	**Target**	**Frequency (MHz)**	**Pressure (MPa)**	**Outcome**
Brugger et al. (2018)	Unknown neuron type	141.9-236.5	2*	Pattern neurite outgrowth
Lin et al. (2018)	Rat hippocampal slices	27.38	0.13–0.32	Evoke action potential and modulate sodium currents
Lin et al. (2019)	Rat hippocampal slices	27.38	0.1*	Modulate potassium current and action potential
Lin et al. (2020)	Human epileptic slices	28, 6.57	0.13	Inhibitory epileptiform discharges
Ye et al. (2018)	Transfected rat hippocampal neuron culture	29.92	0.25–0.45	Evoke action potential
Zhou et al. (2017)	Caenorhabditis elegans	28.11	0.4*	Reverse locomotion and active ASH neurons
Miansari et al. (2019)	Caenorhabditis elegans	19.95	0.01–0.2*	Reduce worm’s mobility and short-term memory

*Converted Values: This work didn’t report the acoustic pressure, but input power or acoustic intensity. The acoustic pressure in this table was converted either from input power or acoustic intensity as follows. To obtain acoustic pressure P_A from intensity I: The relationship between intensity and acoustic pressure is$I=\frac{P_{A}^{2}}{2\,\rho_{l}\,v}$*where P_A is the acoustic pressure amplitude*, ρ_*l*_*is the density of surrounding medium (∼10^3^ kg/m^3^), and v is the sound speed in the medium (∼1,530 m/s)* (Plaksin et al., 2014). *To obtain acoustic intensity from input power: First, the insertion loss is estimated as 3 dBm. The actual SAW power can be estimated as*$P_{S\,A\,W}=\frac{1}{4}\,P_{I\,N}$. *Second, the path width of the SAW can be estimated as the aperture width W of the IDT. As a portion of the energy of the SAW will be coupled into the solution covering the IDT, the SAW energy will decay exponentially along the propagation direction. The SAW energy* 1/*e decay length along the propagation direction (x-direction) can be calculated as*$l_{o\,p\,X}^{C\,a\,l\,c}=12.5\times \lambda_{S\,A\,W\,X}$. *The applied SAW intensity can thus be estimated as*$I=\frac{P_{S\,A\,W}\,\left(1-\frac{1}{e}\right)}{W\times l_{o\,p\,X}^{C\,a\,l\,c}}$ (Stamp et al., 2016).

## Mechanisms of Acoustic Neuromodulation

**FIGURE 1:**
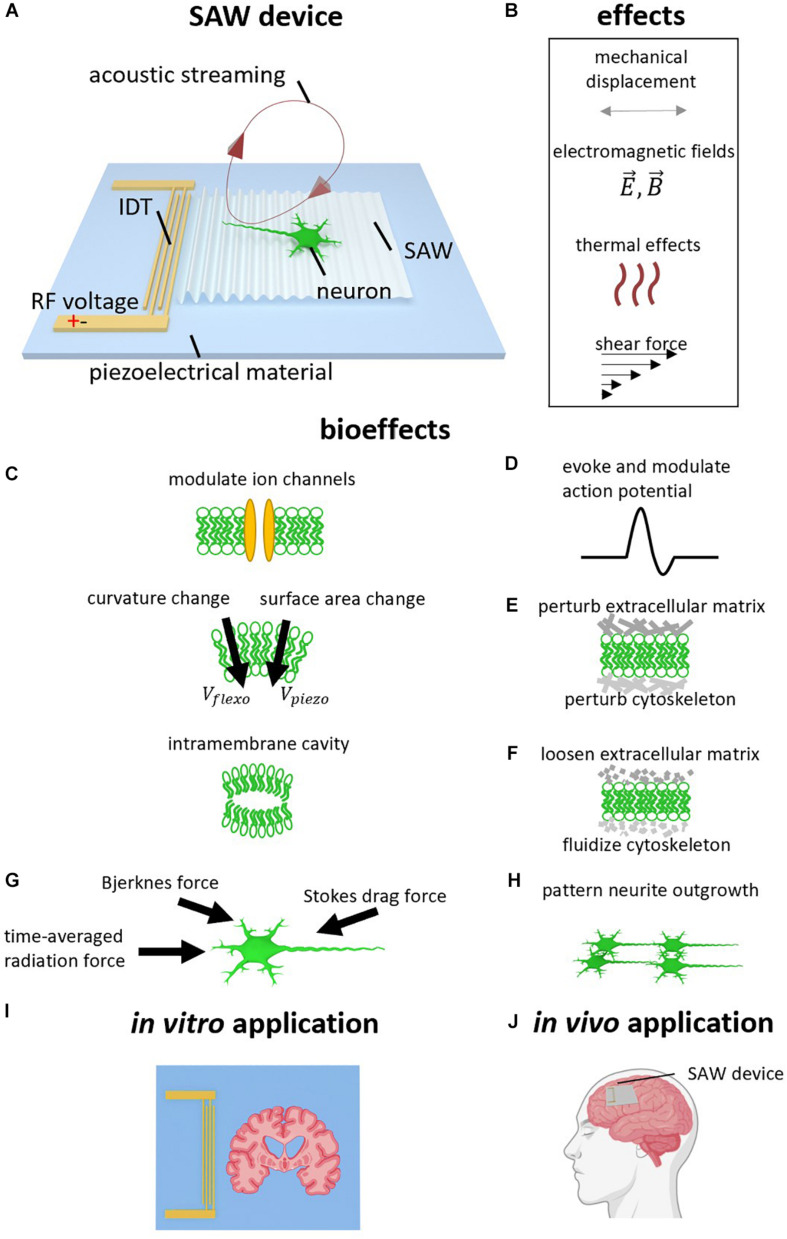
Surface acoustic wave (SAW) neurostimulation mechanisms. **(A)** SAW device. Radio frequency AC voltage is applied to the interdigital transducer (IDT) to generate a SAW which propagates on the surface of the piezoelectric substrate. The SAW refracts the longitudinal wave into the neural medium at the Rayleigh angle. The refracted longitudinal wave results in acoustic streaming. **(B)** Acoustic effects caused by SAW device. In addition to the acoustic vibration, other effects due to the high frequency electromagnetic field, heating, and shear forces may also be considered. **(B–H)** Bioeffects caused by acoustic effects. **(C)** Ion channels can be modulated, and the generated ultrasound can change membrane curvature, surface area, and generate intramembrane cavities. **(D)** Action Potentials (AP) can therefore be evoked and modulated. **(E)** The ultrasound can perturb the extracellular matrix and cytoskeleton. **(F)** Ultrasound can also loosen the extracellular matrix and fluidize the cytoskeleton, analogous to the rejuvenation of soft glassy materials. **(G)** SAW actuation generates acoustic forces including the acoustic radiation force, intercellular Bjerknes force, and the stokes drag force. **(H)** Neurite outgrowth can be patterned via SAW. **(I)** *In vitro* application using SAW device to stimulate brain slice. **(J)** Potential *In vivo* application using wearable or implantable SAW device to treat neurological disorder.

There have been many studies in which bulk-wavemode transducers have been utilized for neuromodulation (Blackmore et al., 2019). Since both these and SAW transducers generate acoustic fields via electrical excitation of a piezoelectric material, with sinusoidal pressure wavefronts resulting in human tissue for both cases, SAW devices are expected to modulate neural activities via similar mechanisms. Therefore, the discussion in this section is primarily based on research using traditional ultrasound transducers (Sassaroli and Vykhodtseva, 2016; Blackmore et al., 2019). SAWs, similar to those generated by traditional ultrasound transducers, can generate four effects: mechanical displacement, electromagnetic fields (EMFs), thermal effects, and acoustic streaming (see Figure 1B), though their relative impact is likely to differ due to higher native SAW frequencies. When a neuron is exposed to SAW (see Figure 1A), these effects may act on ion channels, cell membranes, extracellular matrix, and the cytoskeleton in the cytoplasm. These effects then lead to changes in membrane potentials, activation of action potentials (APs), and altered neurite outgrowth (see Figures 1C–H). Here, we discuss the potential mechanisms of the interaction between these four effects and the cell components separately, even though crosstalk interactions between them are still unclear.

### Mechanical Displacement

One of the effects that ultrasound can induce is mechanical displacement, which results in the periodic movement of all the molecules in targeted neurons. The amplitude of the mechanical displacement ranges from picometers to microns depending on the actuation frequency and signal power (Smagin et al., 2017; Feeney et al., 2019), where these displacements drive ion channel activation by changing the conformational state of channel proteins and their surrounding bilayer (Perozo et al., 2002; Gullingsrud and Schulten, 2004; Jensen and Mouritsen, 2004; Lee, 2004; Phillips et al., 2009; Buyan et al., 2019; Callahan et al., 2019). Existing research using traditional ultrasound has indicated that there are several ion channels that respond to mechanical displacement (Blackmore et al., 2019). These include calcium channels (Tyler et al., 2008), the two-pore-domain potassium family (K2P), including TREK-1, TREK-2, and TRAAK (Kubanek et al., 2016), the Na_*V*_1.5 channels (Kubanek et al., 2016), and the transient receptor potential channels including TRP-4 (Ibsen et al., 2015) and TRPM7 (Li et al., 2018). In the case of SAW actuation, this wavemode has similarly been demonstrated to evoke APs in rat brain slices by altering both sodium and potassium channel kinetics (Lin et al., 2018, 2019). There are also a number of proteins whose native structure renders them mechanosensitive, where these proteins are used to sense pressure, vibration, stretch, and shear stress (Ranade et al., 2015). SAW stimulation of the mechanosensitive channels, such as MscL presented in *Escherichia coli* (Ye et al., 2018) and transfected Piezo 1 channels in human embryonic kidney cells (Liao et al., 2019) have also been demonstrated (see Table 1).

In addition to ion channels, mechanical displacement can also act on cell membranes by changing the membrane configurations elastically (Prieto et al., 2013), including cell membrane thickness and surface area density (Wobschall, 1972; Heimburg, 2012; Gonzalez-Perez et al., 2016), as well as membrane curvature (Petrov, 2002, 2006). It can also lead to the generation of cavities between intramembrane spaces (Krasovitski et al., 2011; Plaksin et al., 2014; see Figure 1C). All these changes may alter the membrane capacitances (Heimburg, 2012) and resistances (Chen et al., 2019). The changes of membrane curvature can further alter the cell membrane potential via the flexoelectric (Petrov and Sokolov, 1986) and piezoelectric (Mosgaard et al., 2014) effects of the cell membrane, in which the membrane surfaces become polarized due to bending and compression, respectively. As a result, this can explain the generation and modulation of APs by ultrasound (Krasovitski et al., 2011; Plaksin et al., 2014; Chen et al., 2019; Jerusalem et al., 2019; see Figure 1D).

A third possible interaction between mechanical displacement and neurons is through the extracellular matrix (ECM) and the cytoskeleton (see Figure 1E). It has been proven that cells are capable of modulating diverse physiological processes by transducing mechanical stimuli and mechanical features of ECM, into biochemical signals (Nourse and Pathak, 2017). For example, Gaub and Muüller (2017) showed that mechanical activation of Piezo1 channels is associated with their surrounding ECM and these Piezo1 channels become less sensitive in the absence of the ECM. In another study, high-intensity ultrasound enabled nanoparticle delivery in tumor tissues by loosening ECM (Lee et al., 2017; see Figure 1F). While both studies used a traditional ultrasound transducer, similar interactions between ECM and SAW are expected.

The cytoskeleton in the cytoplasm is also associated with many vital physiological processes in neurons, including cell adhesion, migration, and neurite outgrowth. Cytoskeletal microtubules may vibrate in response to the mechanical resonance introduced by SAW stimulation (Hameroff et al., 2013; Rafati et al., 2020). Cytoskeletal actin fibers were found to be driven through fluidization during ultrasound operation, and cytoskeleton remodeling in response to ultrasound has been reported (Mizrahi et al., 2012; Zhang et al., 2012; Samandari et al., 2017), where the fluidization and remodeling of the cytoskeleton are analogous to the rejuvenation of soft glassy materials (López-Menéndez and Rodríguez, 2017). Ultrasound has been further shown to inhibit cell adhesion and migration (Alter et al., 1998), enhance cytoskeleton contractility (Fan et al., 2013), retract neurites and shrink cell bodies (Hu et al., 2013), and enhance neurite outgrowth (Ventre et al., 2018). SAW is expected to have a similar impact on cytoskeleton architecture, where SAW has been demonstrated to direct neurite outgrowth into defined patterns (Brugger et al., 2018).

Lastly, the harmonic mechanical displacement can lead to three different forces that act across the entire cell. First is the time-averaged radiation force (Laurell et al., 2007), which comes from the averaged effect of the harmonic vibrating molecules (see Figure 1G). Second is the intercellular force (Bjerknes force) caused by the acoustic wave scattering in the cell’s vicinity (Laurell et al., 2007; Bruus, 2012). Third is the stokes drag force originating from fluid friction (Devendran et al., 2016). These acoustic forces are widely used in microfluidic research for micro/nano particle manipulation (Ding et al., 2013; Go et al., 2017). These acoustic forces, together with the ECM and cytoskeleton remodeling, may contribute to the observation of guided neurite outgrowth mentioned in section “SAW Patterned Neurite Outgrowth” (see Figure 1H).

### High-Frequency Electromagnetic Fields

When a SAW is induced on a piezoelectric substrate, high-frequency electromagnetic fields (EMFs) are also generated, since mechanical displacements produce local electrical potentials; a SAW thus represents a seamless and continuous electro-mechanical coupling as the wavefronts propagate along the substrate. The EMFs have a frequency equal to the MHz–GHz actuation rate, where the electric component of the EMF is in the order of several kV/cm. Such electrical strength can produce cell-based effects in addition to acoustic vibration (Stamp et al., 2016). The biological effects from the EMFs have been shown to be operative in pulsed electromagnetic field therapy (PEMF), which also operates in the MHz range (Binder et al., 1984). PEMF is used for reducing pain (Foley-Nolan et al., 1990; Markov, 2007) and enhances wound healing (Marepalli et al., 2018). Although the mechanism underlying this process is not clear, there is some evidence indicating that EMFs can stimulate microtubules whose electronic resonance also lies in the MHz range (Sahu et al., 2013). Using cultured neuronal cells, MHz range EMFs have also been shown to modulate cytosolic Ca^2+^ concentrations and Ca^2+^ spike rates (Rao et al., 2008).

### Acousto-Thermal Effects

The thermal effect is another possible mechanism that can contribute to SAW neuromodulation. A component of the ultrasound energy will be absorbed by lattice vibrations and defects of piezoelectric materials, and therefore be transferred into heat (Chernov and Roe, 2014; Xu et al., 2019). Previous SAW stimulation experiments commonly report a temperature increase of about 0.1–1.0°C (Stamp et al., 2016; Zhou et al., 2017; Lin et al., 2018, 2019, 2020; Ye et al., 2018; Liao et al., 2019). Neurons are highly sensitive to temperature changes, and even a small temperature increase (<1°C) may lead to changes in action potential (AP) kinetics and ion channel activity (Guttman, 1966; Chapman, 1967; Thompson et al., 1985; Cesare et al., 1999; Maingret et al., 2000; Smith et al., 2002; Xu et al., 2002; Kang et al., 2005; Lee et al., 2005; Black et al., 2016; Kiyatkin, 2019). There are different types of thermosensitive transient receptor potential (TRP) channels such as TRPV1 and TPRV4 on the neuronal membrane, which can be activated at 37°C (Voets et al., 2004; Shibasaki et al., 2007; Hasani et al., 2015). One study showed that the Ca^2+^ influx of TRP channels plays a critical role in the guidance of developing neuronal dendrite cones (Talavera et al., 2008). Ultrasound-induced heating may also perturb membrane capacitance and resistance, and therefore membrane potentials (Carvalho-de-Souza et al., 2015; Hasani et al., 2015; Gulick et al., 2017). Besides, heating can contribute to ECM and cytoskeleton remodeling (Hirunsai et al., 2015).

### Shear Force Induced by Acoustic Streaming

Acoustic streaming is also a vital effect describing vortical fluid motion induced by acoustic waves. This is a second-order effect (compared to the first-order sinusoidal mechanical displacement) arising from both viscous coupling within the acoustic boundary layer and Reynolds stresses induced by the attenuation of the acoustic wavefronts as they propagate through a fluid medium (Lighthill, 1978; Uchida et al., 1995). Acoustic streaming is often used in microfluidic devices, e.g., acoustic mixing, which is to mix fluids and/or particles in microscale channels. Cells subject to this acoustic streaming will experience a shear force due to the inhomogeneous streaming velocity (Djukelic et al., 2017). Although the precise mechanism is unclear, these shear forces can evoke APs (Kugler et al., 2018) and calcium responses (Ravin et al., 2012) in neurons. A recent study revealed that this can also affect cell adhesion and survival rate in cell cultures (Sivanantha et al., 2014; Jötten et al., 2019). This indicates that the shear force generated by acoustic streaming may interact with cells through biological pathways, including the cell membrane, ECM, and cytoskeleton, rather than simply displacing the cells.

## Neuromodulation via Surface Acoustic Waves

In the following sections we summarize recent studies evidencing the bioeffects described in section “Mechanisms of Acoustic Neuromodulation” and Figure 1C. A list of these studies is summarized in Table 1.

### SAW Patterned Neurite Outgrowth

Engineered networks of neurons are a useful tool for understanding connections and communication in neural circuits. Brugger et al. (2018) reported the patterned growth of neurites by applying SSAW in cell culture. They found that, after initial adherence, by applying SSAW to the cultured neurons the majority of neurite outgrowth followed the axis of the SSAW field’s nodal positions (Brugger et al., 2018). This neurite outgrowth patterning originates most likely from the forces generated by the surface deformation due to the SSAW (Laurell et al., 2007), as well as the ECM and cytoskeleton remodeling (see section “Mechanical Displacement”). This study indicates that SSAW provides a promising technology for forming artificial neuronal networks (see Figure 1H).

### SAW Modulated Neural Activities

The majority of neurological disorders are caused by altered functions or mutations in ion channels of neurons (Kumar et al., 2016). Thus, modulating the activities in affected ion channels can be useful to understand the mechanism behind the disease and to find possible treatments.

Lin et al. (2018, 2019) described how SAW can evoke APs by modulating ion channel activities in rat brain slices (see Figure 1I). They performed patch clamping on the neurons from rat hippocampal slices and monitored their membrane potentials and ionic currents. Their results revealed that SAW can activate APs and increase spike rates induced by intracellular current injection. They found that during SAW application, the cells require less current, which would otherwise have to be injected intracellularly to evoke an AP, and the APs kinetics changed with reduced AP half-widths and reduced post-hyperpolarization peak time (Lin et al., 2018, 2019). Applying SAW also decreased the resting membrane potential (RMP), membrane input resistance and membrane voltage time constant (Lin et al., 2018, 2019). By applying channel blockers, Lin et al. (2018) discovered that SAW can modulate sodium channel kinetics. In a follow-up study, they further found that SAW increases potassium efflux. The authors therefore suggested the use of SAW for treating potassium-current-related neurological disorders, such as long QT syndrome and epilepsy (Lin et al., 2019).

The studies described above used continuous SAW, resulting in excitatory effects. Applying a pulsed SAW with a pulse duration of 5 ms and pulse repetition frequency (PRF) of 100 Hz, Lin et al. (2020) found that the pulsed SAW inhibits the epileptiform discharges in both mice and human epileptic brain slices, therefore offering a potential treatment for epilepsy. However, the mechanism behind the different excitatory and inhibitory effects from continuous and pulsed SAW stimulation remains unclear (Lin et al., 2020).

SAW has also been demonstrated to evoke APs via mechanosensitive channels. Ye et al. (2018) demonstrated that SAW evoked APs in rat hippocampal neuronal cultures that had been transfected with the *Escherichia coli* mechanosensitive channel (MscL). They showed that the presence of MscL is essential for AP activation by SAW. In addition, they found that, matters, such as Calcein fluorescent dye, can pass the cell membrane through the opening of the MscL during activation, indicating potential application of drug delivery using SAW (Ye et al., 2018). While, in another study by Lin et al. (2018), in which APs in rat hippocampal slices were evoked without MscL transfection. This suggested that SAW can also activate native mechanosensitive channels. Further research is required to fully understand how SAW evokes APs.

SAW also affects microorganisms, e.g., Caenorhabditis elegans (C. elegans), one of the simplest multicellular organisms with a well-studied nervous system. Zhou et al. (2017) found that C. elegans’ locomotion can be reversed on-demand by SAW stimulation. Extending the duration of SAW stimulation and exposing larger proportions of the worm’s bodies resulted in a greater duration of reversal (Zhou et al., 2017). By imaging calcium ion activities, they observed calcium transients in the C. elegans ASH neurons, a type of sensory neuron, immediately after SAW stimulation, suggesting that SAW may reverse the locomotion of C. elegans by actuating their sensory neurons. In the same study, Zhou et al. (2017) further showed that the majority of the thermosensory AFD neurons were not activated during SAW stimulation. This indicates that the activation of the C. elegans was mainly caused by mechanical cues rather than heat (Zhou et al., 2017). While Zhou et al. (2017) used single-shot, short-pulsed (6.40 ms pulse) SAW, Miansari et al. (2019) found the C. elegans can be paralyzed when applying a 10 s continuous SAW-driven stimulation, and their mobility and short-term memory was also reduced. The reduced mobility was found mainly due to mechanical displacement rather than acoustic streaming (Miansari et al., 2019).

## Conclusion and Future Perspectives

Miniaturized SAW devices are finding increasing use as a mechanical stimulating tool for neuronal stimulation. Compared to traditional ultrasound transducers, they demonstrate less self-heating and offer a higher power efficiency, thus presenting a safer method to stimulate neurons and investigate neuronal processes. By virtue of both the smaller dimensions of these devices and their higher frequencies, they also offer the opportunity for highly targeted neuromodulation. Previous studies showed that SAW-driven ultrasound can pattern neurite outgrowth and modulate neural activities, revealing a potential to apply SAW as a therapy to treat neurological disorders.

Different SAW device configurations, including different IDT structures and materials, may also find use in future applications. There are three primary IDT configurations: uniform, focused, and slanted (tapered) IDTs. Current SAW devices for neuromodulation mainly use uniform or focused IDTs, which can only stimulate a restricted area on the device. In comparison, it is possible to stimulate arbitrary locations using slanted IDTs (Ding et al., 2012; Khalid et al., 2013; Naseer et al., 2017). If neurons are cultured on these devices, acoustic stimulation would only act on a localized and controllable area. Most SAW devices use stiff materials such as LiNbO_3_, ZnO, or AlN as the piezoelectric substrates. The development of future implantable and wearable devices for *in vivo* applications will benefit from the use of flexible piezoelectrics, e.g., Polyvinylidene fluoride (PVDF) or ZnO thin films on metallic foils (Jin et al., 2013).

Currently, most studies using traditional ultrasound transducers are conducted either *in vitro* or *in vivo* (Fini and Tyler, 2017), whereas SAW devices are only used for *in vitro* experiments (see Table 1). Expanding the use of SAW to *in vivo* applications, however, may offer advantages in certain therapeutic applications. By virtue of their dimensions, power efficiency and targetability, this has the potential for use in wearable and implantable wireless ultrasonic devices (see Figure 1J), e.g., SAW-brain implants enhanced drug delivery through the blood-brain barrier (BBB) (Mainprize et al., 2019), neuronal growth after neural injury (Stamp et al., 2016) and neuromodulation for treating neurological disorders (Leinenga et al., 2016; Lin et al., 2020).

## Author Contributions

DP wrote the first draft of the manuscript, addressed, and coordinated all comments and revisions. WT, MS, and DC gave the comments and contributed to manuscript revision. MI and SP gave the final comments and suggestions. All authors approved the submitted version.

## Conflict of Interest

SP was a shareholder and public officer of Carbon Cybernetics Pty Ltd., a company developing diamond and carbon-based medical device components. SP was a shareholder in iBIONICS, a company developing a diamond based retinal prosthesis. The remaining authors declare that the research was conducted in the absence of any commercial or financial relationships that could be construed as a potential conflict of interest.
